# Identification and validation of hub genes and molecular classifications associated with chronic myeloid leukemia

**DOI:** 10.3389/fimmu.2023.1297886

**Published:** 2024-01-12

**Authors:** Fangmin Zhong, Fangyi Yao, Shuai Xu, Jing Zhang, Jing Liu, Xiaozhong Wang

**Affiliations:** Jiangxi Province Key Laboratory of Laboratory Medicine, Jiangxi Provincial Clinical Research Center for Laboratory Medicine, Department of Clinical Laboratory, The Second Affiliated Hospital, Jiangxi Medical College, Nanchang University, Nanchang, Jiangxi, China

**Keywords:** chronic myeloid leukemia, WGCNA, hub gene, diagnosis, biomarker

## Abstract

**Background:**

Chronic myeloid leukemia (CML) is a kind of malignant blood tumor, which is prone to drug resistance and relapse. This study aimed to identify novel diagnostic and therapeutic targets for CML.

**Methods:**

Differentially expressed genes (DEGs) were obtained by differential analysis of the CML cohort in the GEO database. Weighted gene co-expression network analysis (WGCNA) was used to identify CML-related co-expressed genes. Least absolute shrinkage and selection operator (LASSO) regression analysis was used to screen hub genes and construct a risk score model based on hub genes. Consensus clustering algorithm was used for the identification of molecular subtypes. Clinical samples and *in vitro* experiments were used to verify the expression and biological function of hub genes.

**Results:**

A total of 378 DEGs were identified by differential analysis. 369 CML-related genes were identified by WGCNA analysis, which were mainly enriched in metabolism-related signaling pathways. In addition, CML-related genes are mainly involved in immune regulation and anti-tumor immunity, suggesting that CML has some immunodeficiency. Immune infiltration analysis confirmed the reduced infiltration of immune killer cells such as CD8+ T cells in CML samples. 6 hub genes (LINC01268, NME8, DMXL2, CXXC5, SCD and FBN1) were identified by LASSO regression analysis. The receiver operating characteristic (ROC) curve confirmed the high diagnostic value of the hub genes in the analysis and validation cohorts, and the risk score model further improved the diagnostic accuracy. hub genes were also associated with cell proliferation, cycle, and metabolic pathway activity. Two molecular subtypes, Cluster A and Cluster B, were identified based on hub gene expression. Cluster B has a lower risk score, higher levels of CD8+ T cell and activated dendritic cell infiltration, and immune checkpoint expression, and is more sensitive to commonly used tyrosine kinase inhibitors. Finally, our clinical samples validated the expression and diagnostic efficacy of hub genes, and the knockdown of LINC01268 inhibited the proliferation of CML cells, and promoted apoptosis.

**Conclusion:**

Through WGCNA analysis and LASSO regression analysis, our study provides a new target for CML diagnosis and treatment, and provides a basis for further CML research.

## Introduction

Chronic myeloid leukemia is a malignant tumor that affects the blood and bone marrow (1). It is mainly induced by the BCR-ABL1 fusion gene, which encodes a protein with strong tyrosine kinase activity and activates various signaling pathways (2). At present, the main therapeutic drugs for CML are tyrosine kinase inhibitors (TKIs) targeting BCR-ABL1 (2). The development of the first-generation TKI imatinib (IM) has changed the treatment status of CML, and the prognosis of patients has been significantly improved (3). It is widely used and has a good therapeutic effect. However, due to the existence of escape mechanisms, tumor cells often develop resistance to kinase drugs, leading to the malignant progression of the disease, which seriously affects the health of patients (4). In addition, the long-term use of TKI will also produce many complications, affecting the quality of life of patients (5). Therefore, there is an urgent need to identify novel molecular targets for the diagnosis and treatment of CML.

With the progress and development of sequencing technology, bioinformatics has been widely used to explore the genetic changes of tumors, and to find new targets for early diagnosis and therapeutic intervention of tumors. The Gene Expression Omnibus (GEO) database contains gene expression profiles of various diseases and tumor samples and corresponding clinical information, which can be used for in-depth analysis (6). Weighted gene co-expression network analysis (WGCNA) is a bioinformatics tool to screen genes with similar expression patterns related to disease phenotypes by constructing free-scale gene co-expression networks (7). The reliability of this method has been widely verified (8–10), and to a large extent, it overcomes the limitations caused by only focusing on differentially expressed genes (DEGs). Therefore, hub genes that are highly correlated with clinical phenotypes can be defined as potential biomarkers and therapeutic targets.

In this study, we systematically analyzed the CML dataset GSE13159 in the GEO database, combined with differential expressed expression analysis and WGCNA analysis, identified a set of co-expressed genes significantly associated with CML, and determined the biological functions of these genes by enrichment analysis. Subsequently, the least absolute shrinkage and selection operator (LASSO) analysis was used to screen out signature genes that had high diagnostic value for CML and could predict treatment response in CML patients. We also identified two molecular subtypes with distinct immune landscapes based on hub gene expression. Finally, the diagnostic performance of the risk score model constructed by hub genes was further improved. These signatures were validated using an additional public cohort and our clinical real-world cohort. Therefore, these findings will help reveal more underlying mechanisms of CML, as well as the potential value of these targets in CML treatment.

## Materials and methods

### Data acquisition and processing

We downloaded the CML data sets (GSE13159, GSE144119) from the GEO database. GSE13159 contains 76 CML samples and 74 normal samples, and we normalized the original “cel” files. GSE144119 contained 48 newly diagnosed CML samples and 32 remission CML samples, as well as 17 normal samples, and the data were converted to transcripts per kilobase million (TPM) values for subsequent analyses. GSE13159 was used as the analysis cohort, and GSE144119 was used for subsequent validation. The normalized RNA-seq data (TPM values) of 173 TCGA-LAML (The Cancer Genome Atlas-Acute Myeloid Leukemia) samples containing clinical information were downloaded from the UCSC XENA database (https://xenabrowser.net/datapages/).

### Pathway activity assessment and function enrichment analysis

The gene set variation analysis (GSVA) algorithm was used to calculate the enrichment score of the gene set to quantify the activity of the corresponding biological process or signaling pathway. The GSVA score was calculated based on the overall position of the gene set genes in the expression ranking of all genes, and the higher the overall expression level of these genes, the higher the GSVA score. KEGG enrichment analysis was used to analyze the function of phenotypic-related genes identified by WGCNA. We perform these analyses in the “clusterProfiler” package (11).

### Analysis of immune cell infiltration

CIBERSORT algorithm based on support vector regression analysis was used to analyze the infiltration proportion of 22 kinds of immune cells in CML samples (12).

### Weighted correlation network analysis

WGCNA is a tool for assessing gene expression correlations and visualizing co-expression networks. The “WGCNA” software package was used to identify CML-related genes in the GSE13159 cohort. Pearson correlation analysis was used to form an adjacency matrix for all matched genes, and the scale-free topology of the adjacency matrix was realized based on the optimal soft threshold power. Then, we further transform the adjacency matrix into a topological overlap matrix (TOM). Based on the TOM difference measure, the minimum module size was set to 30, the cutting height was set to 0.2, and the genes with similar expression patterns were divided into the same modules through average linkage hierarchical clustering. Then, the correlation between module characteristic genes (MEs) and CML was assessed, and the modules that met the purpose of the study were determined according to the degree of correlation.

### Identification of DEGs between normal and CML samples

The empirical Bayesian approach via the “limma” package was used to determine DEGs between normal and CML samples (13). Genes with adjusted P-values < 0.05 and |logFC| > 1 were considered significantly different.

### Construction of risk score model

Overlapping genes of CML-related genes and DEGs identified by WGCNA were used for the identification of CML hub genes. Then, the LASSO regression algorithm was used for dimensionality reduction analysis to screen out the most related genes with CML (14). In addition, based on the correlation of hub genes, LASSO regression analysis assigned a coefficient to each gene, and the expression of each gene was multiplied by its coefficient and added to obtain a risk score, which was used to analyze the diagnostic value of the combination of hub genes in CML. Risk score = NME8 × 1.160 + DMXL2 × 0.853 + CXXC5 × -0.126 + SCD × 0.610 + FBN1 × 0.405, where gene ID refers to the expression value of each gene.

### Identification of molecular subtypes based on hub genes

Consensus cluster analysis was performed to identify CML molecular subtypes based on hub gene expression using the “consensusclusterplus” package. Clustering was performed for 1000 iterations to ensure reliable and stable results. t-distributedstochastic neighbor embedding (t-SNE) was used to validate the classification.

### Construction of competing endogenous RNA network

Target miRNAs of hub genes were found in the miRTarBase, miRDB, and TargetScan databases. Perl programming language was used to perform the prediction analysis of the target lncRNAs of these miRNAs in the miRcode database.

### Prediction of treatment response for different molecular subtypes

The half-maximal inhibitory concentrations (IC_50_) of different CML samples to therapeutic drugs were predicted based on drug response data of blood cell lines from the Cancer Genome Project (CGP) database (https://cancer.sanger.ac.uk/cosmic) via the “pRRophetic” package. Tumor Immune Dysfunction and Exclusion (TIDE, http://tide.dfci.harvard.edu/) was considered a good predictor of immunotherapeutic response for molecular subtypes.

### Clinical sample collection

CML samples and normal samples were collected for sequencing analysis in accordance with the Declaration of Helsinki and institutional guidelines, and informed consent was obtained from each patient and healthy volunteer who had undergone the appropriate workup. Our study was approved by the Ethics Committee of the Second Affiliated Hospital of Nanchang University, and sample processing was performed according to the norms. We collected samples from 5 untreated patients with newly diagnosed CML and 5 normal samples from healthy volunteers. The methods and details of sample collection, next-generation sequencing, and processing procedures were described in our previous report (15). Moreover, peripheral blood samples from 15 CML patients and 15 normal controls were collected for quantitative real-time polymerase chain reaction (RT-qPCR) assay to detect hub gene expression. RT-qPCR was performed using a Japanese TAKARA kit on an ABI7500 instrument. The primers are shown in **Supplementary Table S1**.

### Cell culture and detection of cell proliferation and apoptosis

The CML cell line K562 was cultured in RPMI1640 medium supplemented with 10% fetal bovine serum and 1% penicillin-streptomycin in a humidified atmosphere incubator at 37°C with 5% CO_2_. Two different siRNAs targeting LINC01268 (si-LINC01268) and control siRNA (si-NC) were procured from Ribobio (China) and transfected into K562 cells using Lipofectamine 3000 (Thermofisher Scientific) (**Supplementary Table S1**). RT-qPCR was employed to assess the transfection efficiency. Cell proliferation was evaluated using the Cell Counting Kit-8 (CCK-8). For the CCK8 assay, a total of 2×10^4^ cells from various treatment groups were seeded in individual wells of a 96-well plate, with each group being repeated five times. Subsequently, at time points of 0, 24, 48, and 72 hours, respectively, 10 μl of CCK8 solution was added to each well. After incubation at 37°C for two hours, the optical density (OD) value at a wavelength of 450 nm was measured using a microplate reader. Apoptosis assays were performed by staining the cells with Annexin V-PE/7-AAD Apoptosis Assay Kit followed by analysis on a flow cytometer.

### Statistical analysis

We performed the Wilcoxon rank sum test and the Kruskal-Wallis test to determine differences between two or more groups, respectively. The “survminer” package divides patients into high- and low-gene expression groups based on the cut-off point at the minimum p-value of the log-rank test, and the Kaplan-Meier survival curve analysis was used to analyze survival differences between the two groups. The receiver operating characteristic (ROC) curve was used to analyze the diagnostic efficacy of genes. A two-sided P value < 0.05 was considered statistically significant.

## Results

### CML-related genes were identified by WGCNA analysis

**Figure 1:**
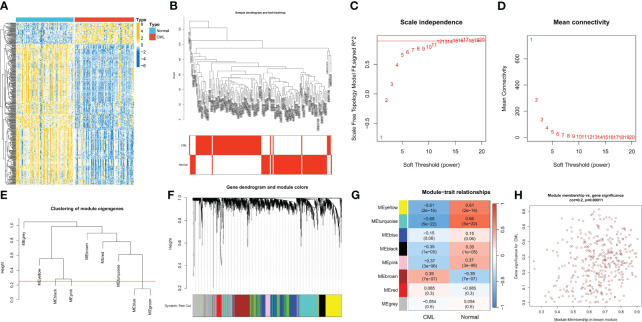
Identification of CML-related genes. **(A)** The heatmap shows differentially expressed genes (DEGs) between CML and normal samples. **(B)** Clustering dendrogram of CML and normal samples. **(C, D)** Scale-free fit index **(C)** and average connectivity **(D)** analysis of various soft threshold powers. **(E)** the cluster of module feature genes. The red line indicates the cutting height (0.25). **(F)** Dendrogram of clustering based on different measures (1-TOM). **(G)** Heatmap of correlation between module genes and phenotypes. Each cell contains a p-value and a correlation coefficient. **(H)** Scatter plot of module characteristic genes associated with CML samples in brown module.

We first performed differential expression analysis between CML and normal samples and obtained a total of 378 DEGs. Heatmap analysis showed that more DEGs were down-regulated in CML (**Figure 1A**). We further performed WGCNA analysis to identify more CML-related genes. The cluster tree diagram showed the clustering characteristics of the samples, and the CML samples had a high degree of discrimination from the normal samples (**Figure 1B**). **Figures 1C, D** show the scale-free fit exponent and average connectivity analysis for various soft threshold powers. We set cut height = 0.25 to merge the blue and green module feature genes (**Figure 1E**). According to the optimal soft threshold power β = 12(unscaled R2 = 0.9), the 5000 genes with the highest standard deviation were divided into eight independent co-expression modules (**Figure 1F**). The correlogram of module-trait relationships showed that the brown module, which contains 369 genes, had the highest correlation with CML (**Figures 1G, H**) (**Supplementary Table S1**). We also found that the blue, green, yellow, black, and pink modules were negatively correlated with CML, and these results were associated with the downregulated expression of most genes in CML.

### Functional analysis of CML-related genes

**Figure 2:**
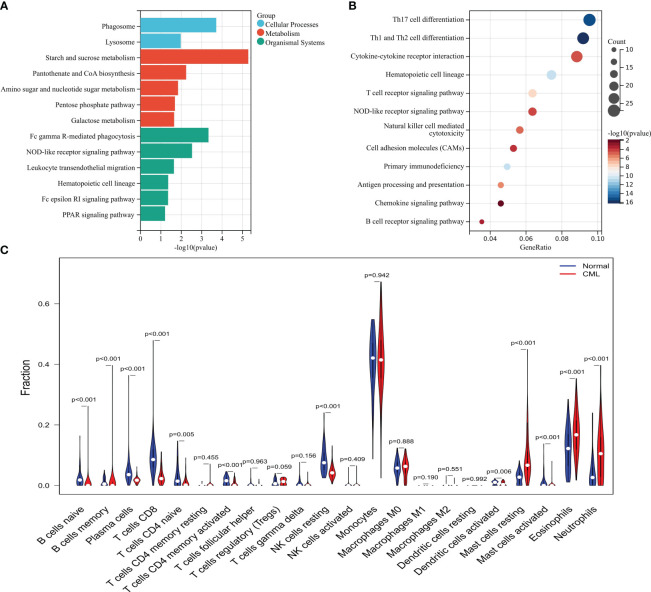
Functional analysis of CML-related genes and immune infiltration analysis. **(A)** KEGG enrichment analysis of brown module genes. **(B)** KEGG enrichment analysis of genes in yellow and turquoise modules. **(C)** Differences in infiltration of 22 immune cells between CML and normal samples.

The brown module genes were mainly related to metabolic-related signaling pathways such as Starch and sucrose metabolism, Pantothenate and CoA biosynthesis, Amino sugar and nucleotide sugar metabolism, Pentose phosphate pathway, and Galactose metabolism (**Figure 2A**). While yellow and turquoise module genes were negatively associated with CML, these genes were mainly enriched in immune-related signaling pathways such as Th17 cell differentiation, Th17 cell differentiation, Cytokine-cytokine receptor interaction, and Hematopoietic cell lineage, T cell receptor signaling pathway, NOD-like receptor signaling pathway, Natural killer cell mediated cytotoxicity (**Figure 2B**). These results indicate that CML has stronger metabolic activity and some immunodeficiency. Immune infiltration analysis showed that CML samples had fewer naive and memory B cells, plasma cells, CD8+ T cells, naive CD4+ T cells, activated memory CD4+ T cells, resting NK cells, and activated dendritic cells, and contained more regulatory T cells (Tregs) than normal samples (**Figure 2C**), which confirm the immunosuppressive features evident in CML samples.

### Identification of CML hub genes

**Figure 3:**
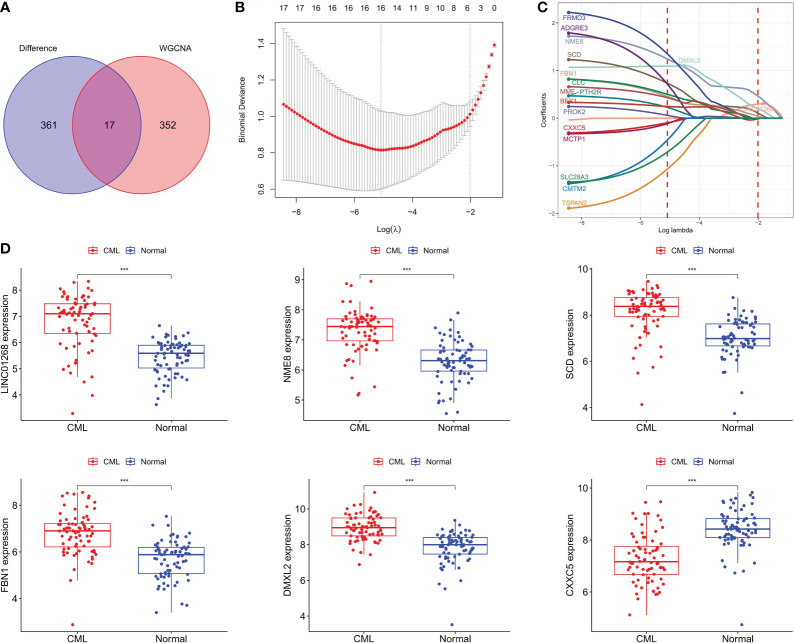
Identification of CML hub genes. **(A)** The intersection of DEGs and brown module genes in WGCNA. **(B)** The penalty coefficient of the minimum 10-fold cross-validation error point was calculated to determine the hub genes. **(C)** determination of hub gene coefficients. **(D)** Differences in the expression of hub genes between CML and normal samples. ***p < 0.001.

We intersected DEGs and WGCNA brown module genes and obtained 17 overlapping genes (**Figure 3A**), and the correlation coefficients of these genes with the brown module in WGCNA and with CML samples were greater than 0.4 (**Supplementary Table S2**), indicating that they were significantly positively correlated with both CML and brown module. LASSO regression analysis further reduced the dimension and screened out 6 hub genes most related to CML, which were LINC01268, NME8, DMXL2, CXXC5, SCD, and FBN1 (**Figures 3B, C**). Boxplots showed that LINC01268, NME8, DMXL2, SCD, and FBN1 were significantly up-regulated and CXXC5 was significantly down-regulated in CML samples compared with normal samples (**Figure 3D**).

### Diagnostic value and prognostic correlation of CML hub genes

**Figure 4:**
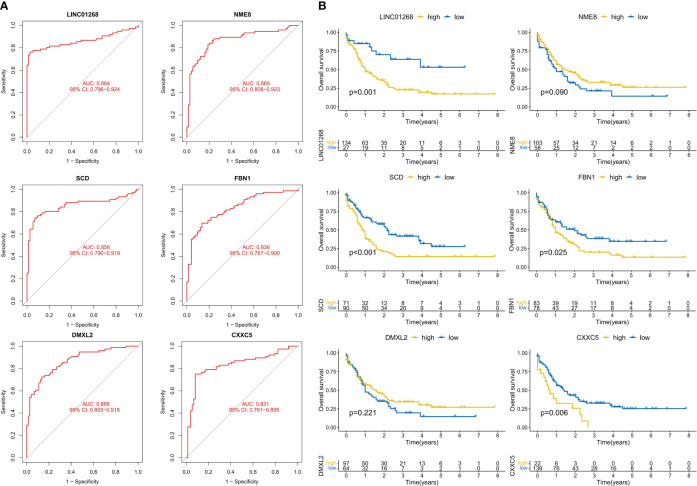
Analysis of the diagnostic and prognostic value of hub genes. **(A)** ROC curve analysis of hub genes. **(B)** K-M curve analysis of hub genes.

We further analyzed the predictive value of CML hub genes for CML. ROC curve analysis showed that all 6 hub gens had high AUC values for the diagnosis of CML, among which LINC01268 was 0.864 (95%CI: 0.796-0.924), NME8 was 0.869 (95%CI: 0.808-0.924), DMXL2 was 0.866 (95%CI: 0.805-0.91), CXXC5 was 0.831 (95%CI: 0.761-0.895), SCD was 0.856 (95%CI: 0.790-0.919), and FBN1 was 0.836 (95%CI: 0.767-0.900) (**Figure 4A**). In addition, considering that approximately 70% of CML cases in blast crisis progress to AML, we analyzed the prognostic predictive value of 6 hub genes in the TCGA-AML cohort. High expression groups of LINC01268, SCD, FBN1, and CXXC5 had significantly shorter overall survival than their low expression groups, respectively, while high expression groups of NME8 and DMXL2 showed better prognosis, but there was no statistical difference (**Figure 4B**).

### Validation of the diagnostic value of CML hub genes

**Figure 5:**
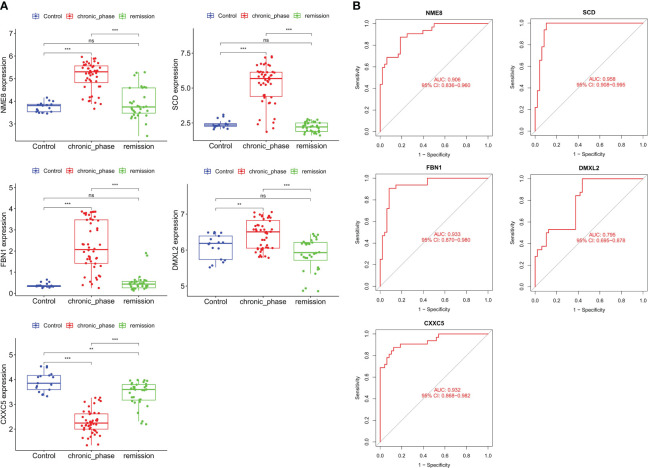
Validation of the expression and diagnostic value of hub genes in the validation cohort. **(A)** Differences in the expression of hub genes in normal samples, newly diagnosed CML samples, and treatment-remission samples. **(B)** ROC curve analysis of hub genes. **p < 0.01; ***p < 0.001; ns, no significance.

**Figure 6:**
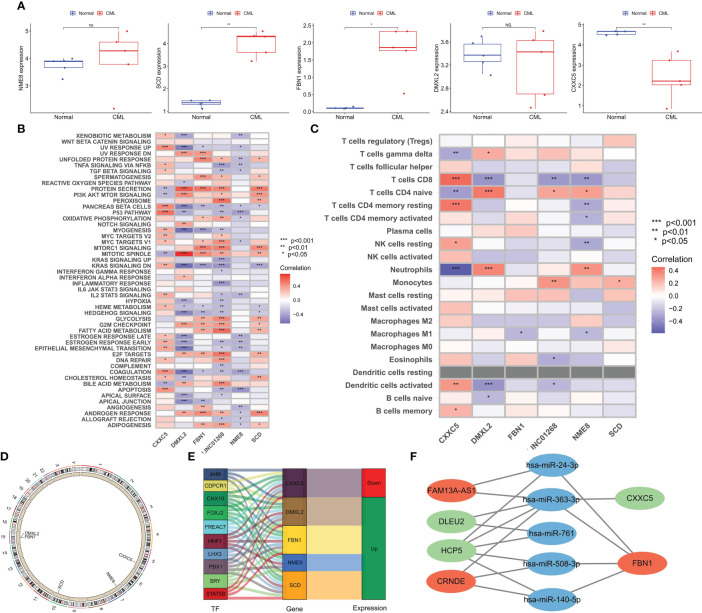
Expression Validation of hub genes in clinical cohort and biological function analysis. **(A)** Differences in the expression of hub genes between CML and normal samples in a clinical cohort. **(B)** Correlation analysis of hub genes and tumor marker pathway activity. **(C)** Correlation analysis of hub genes, and immune cell infiltration. **(D)** The location of hub genes on chromosomes. **(E)** Transcription factors with potential regulatory effects on hub genes expression. **(F)** CeRNA networks with potential regulatory effects on hub genes expression. *p < 0.05; **p < 0.01; ns, no significance.

The GSE144119 cohort contains samples from newly diagnosed and treatment-remission CML. Encourageously, the results of the differential analysis were consistent with the GSE13159 cohort, in which NME8, DMXL2, SCD, and FBN1 expression was significantly increased and CXXC5 expression was significantly decreased in newly diagnosed (chronic phase) CML patients (The expression of LINC01268 was not detected). These hub genes also had predictive value for CML treatment remission. The expression levels of NME8, DMXL2, SCD, and FBN1 were significantly decreased in CML treatment-remission patients, while the CXXC5 expression level was significantly increased, and they all returned to normal control levels. ROC curve analysis confirmed the diagnostic value of these hub genes in CML (**Figure 5A**). The AUC values of NME8, SCD, FBN1, DMXL2, and CXXC5 were 0.906 (95% CI: 0.836-0.960), 0.958 (95% CI: 0.908-0.995), 0.933 (95% CI: 0.870-0.980), 0.795 (95% CI: 0.695-0.878), and 0.932 (95% CI: 0.868-0.982), respectively (**Figure 5B**). In our clinical cohort, we confirmed that SCD and FBN1 expression was significantly upregulated CXXC5 was significantly downregulated in CML, and NME8 and DMXL2 expression were not significantly different due to the small sample size (**Figure 6A**).

### Potential biological mechanisms of CML hub genes

To better explore the biological functions of CML hub genes, we analyzed their correlation with tumor marker pathway activity and immune cell infiltration. CXXC5 expression was related to P53 PATHWAY, DNA REPAIR, MYC TARGETS, and APOPTOSIS, and may be involved in the regulation of CML cell proliferation. DMXL2 was positively correlated with cell cycle-related pathways such as MITOTIC SPINDLE, and G2M CHECKPOINT. FBN1, LINC01268, and SCD were related to the metabolic pathway activity of MTORC1 SIGNALING, GLYCOLYSIS, FATTY ACID METABOLISM, ADIPOGENESIS (**Figure 6B**). The expression of NME8 was negatively correlated with the activity of most tumor marker pathways. In addition, CXXC5 expression was positively correlated with infiltration of CD8+ T cells, resting memory CD4+ T cells, resting NK cells, activated dendritic cells, and memory B cells, suggesting that CXXC5 may be involved in CML anti-tumor immunity (**Figure 6C**). **Figure 6D** shows the location of five hub genes in chromosomes. In addition, we identified a group of transcription factors with potential regulatory effects on hub genes (**Figure 6E**). According to the construction of the CeRNA network (**Figure 6F**), lncRNA FAM13A-AS1 with upregulated expression may promote the expression of FBN1 by competitively binding hsa-miR-24-3p and hsa-miR-363-3p. lncRNA CRNDE may promote the expression of FBN1 by binding hsa-miR-363-3p, hsa-miR-508-3p and hsa-miR-140-5p. The downregulation of lncRNAs DLEU2 and HCP5 may reduce the binding of miR-363-3p, thereby inhibiting the expression of CXXC5.

### The construction of a risk score model can further improve the diagnostic value of hub genes

**Figure 7:**
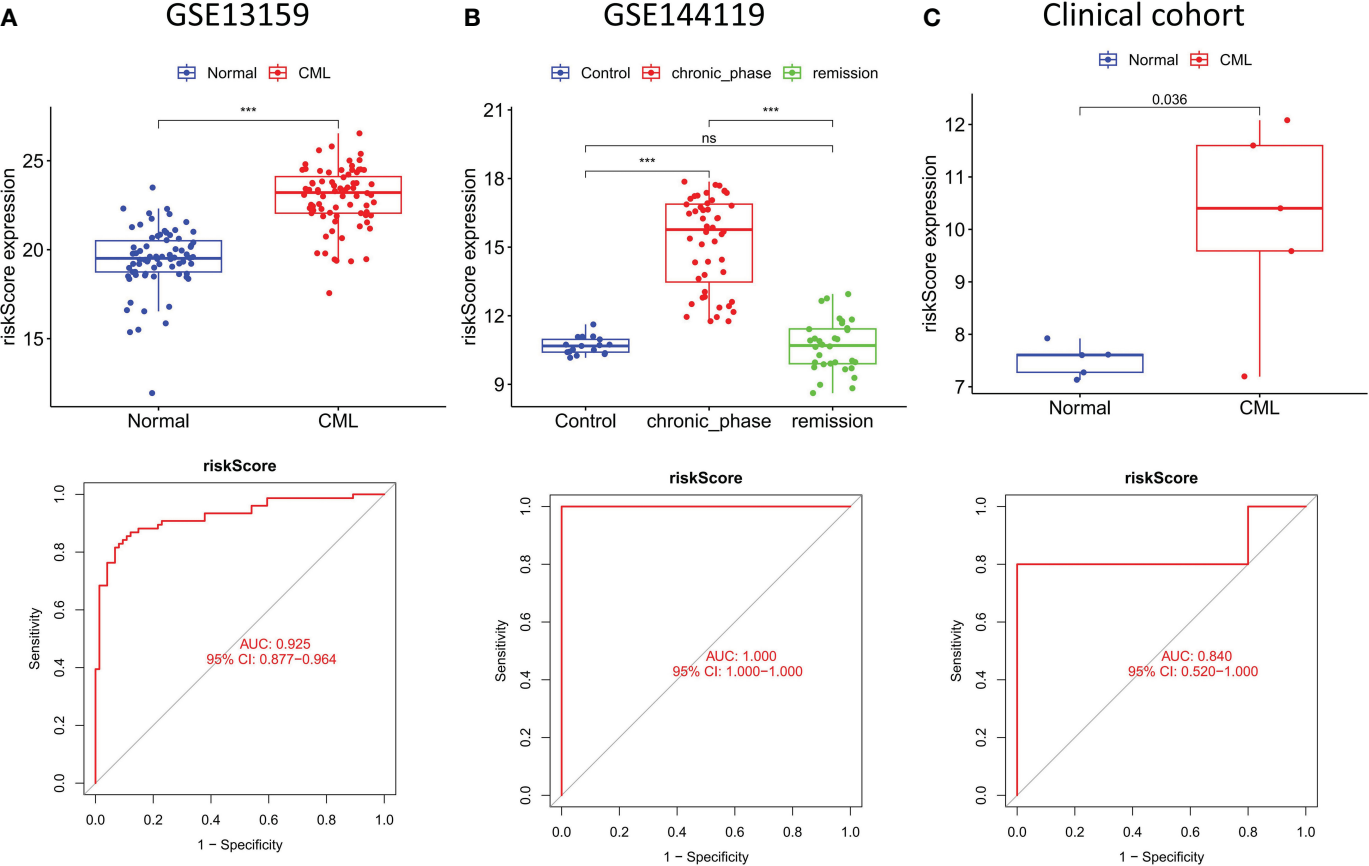
Construction and validation of risk score model. **(A–C)** Expression characteristics and diagnostic value of risk scores in the three cohorts. ***p < 0.001; ns, no significance.

To better improve the diagnostic value of hub genes, we used LASSO regression analysis to construct a risk score model for 5 genes shared by the three cohorts. All three cohorts observed significantly higher risk scores in CML samples than in normal samples, and risk scores in patients in remission tended to be normal (**Figures 7A–C**). ROC curve analysis showed that the diagnostic AUC values in the GSE13159 cohort, GSE144119 cohort, and clinical cohort were 0.925 (95% CI: 0.877-0.964), 1.000 (95% CI: 1.000-1.000) and 0.840 (95% CI: 0.520-1.000), respectively, confirming that the diagnostic value of risk score of hub genes combination was further improved.

### Molecular subtypes identified based on hub genes and prediction of treatment response

**Figure 8:**
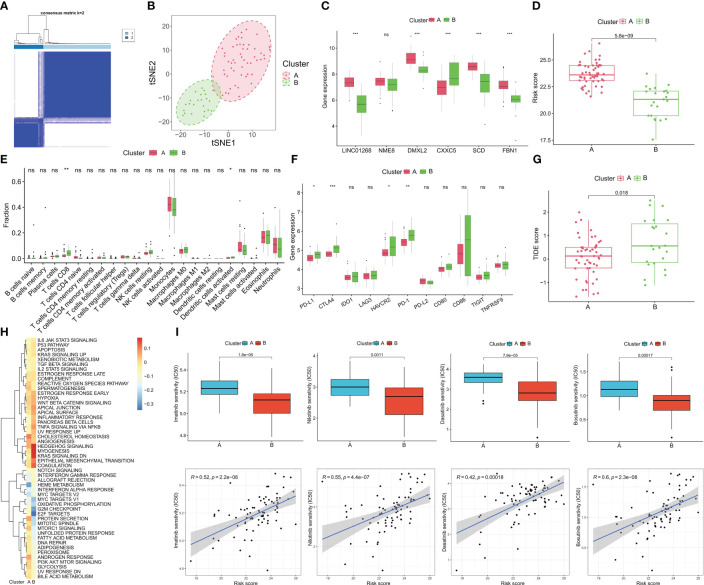
Identification of molecular subtypes of CML and prediction of drug response in different subtypes. **(A)** The consensus clustering algorithm divided CML patients into two different molecular subtypes based on the expression of hub genes. **(B)** t-SNE algorithm was used to verify the classification reliability of the two molecular subtypes. **(C–F)** Differences in expression of DEGs **(C)**, risk score **(D)**, infiltration of 22 immune cells **(E)**, expression of immune checkpoints **(F)**, TIDE scores **(G)**, and activity of tumor hallmark gene sets **(H)** between the two molecular subtypes. **(I)** Differences in therapeutic sensitivity of the two molecular subtypes to four TKIs. *p < 0.05; **p < 0.01; ***p < 0.001; ns, no significance.

We performed cluster analysis of CML samples based on hub gene expression and identified two distinct molecular subtypes (Cluster A and Cluster B) (**Figure 8A**). The t-SNE algorithm verified the reliability of the clustering (**Figure 8B**). Compared with Cluster B, LINC01268, DMXL2, SCD, and FBN1 were up-regulated and CXXC5 was down-regulated in Cluster A (**Figure 8C**). Cluster A also had a significantly higher risk score than Cluster B (**Figure 8D**). Immune infiltration analysis showed that the infiltration levels of CD8+ T cells and activated NK cells were significantly higher in Cluster B than in Cluster A (**Figure 8E**). The expression of immune checkpoints PD-L1, CTLA4, HAVCR2, and PD-1 was also significantly up-regulated in Cluster B (**Figure 8F**). In addition, the TIDE score of Cluster B was significantly higher than that of Cluster A (**Figure 8G**), indicating significant immunosuppression in Cluster B. We also compared the activity of tumor-marker gene sets in the two molecular subtypes (**Figure 8E**). We found metabolic and cell proliferation-related pathways such as MYC targets V1, oxidative phosphorylation, G2M checkpoint, E2F targets, mTORC1 signaling and fatty acid metabolism were more active. In Cluster B, the enrichment scores of cancer-promoting pathways such as hedgehog, epithelial-mesenchymal transition, and TNFA signaling via NFKB were higher (**Figure 8H**). We then predicted the response of different molecular subtypes to TKIs commonly used for CML treatment, and the results showed that Cluster B patients had higher therapeutic sensitivity to imatinib, nilotinib, bosutinib, and dasatinib. Moreover, there was a significant positive correlation between the risk score and the IC_50_ of the four drugs, that is, the higher the risk score, the less sensitive the treatment to the four drugs (**Figure 8I**).

### Expand clinical sample size to validate the expression of hub genes and confirm the oncogenic role of LINC01268

**Figure 9:**
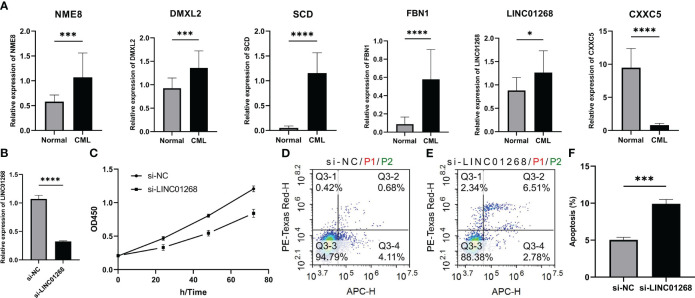
Expression characteristics of hub genes and its relationship with malignant phenotypes of CML cells. **(A)** Differences in mRNA expression of hub genes in peripheral blood samples from 15 CML patients and 15 normal controls. **(B)** mRNA expression level of LINC01268 in K562 cells in LINC01268 knockdown group (si- LINC01268) and control group (si-NC). **(C)** Absorbance at 450nm wavelength after CCK8 treatment in different LINC01268 treatment groups at different time nodes. **(D–F)** Apoptosis levels in different LINC01268 treatment groups. (*P < 0.05; *** P < 0.001; **** P < 0.0001).

The expression of hub genes was validated by RT-qPCR in expanded clinical samples. Encouragingly, the results also confirmed that LINC01268, NME8, DMXL2, SCD, and FBN1 were up-regulated while CXXC5 was down-regulated in CML samples (**Figure 9A**). Previous studies have shown that DMXL2, NME8, and FBN1 primarily exert oncogenic roles through mutations and splice variants (16–18); moreover, the role of SCD in CML has also been reported previously (19). Therefore, we chose to initially explore the biological function of LINC01268 in CML cells. The expression of LINC01268 was significantly inhibited by siRNA (**Figure 9B**). CCK8 assay showed that compared with the si-NC group, the proliferation ability of CML cells in the si-LINC01268 group was significantly reduced (**Figure 9C**). Moreover, the apoptosis rate of the si-LINC01268 group was higher than that of the si-NC group (**Figures 9D–F**). These results reveal the oncogenic role of LINC01268 and its potential as a therapeutic target for CML.

## Discussion

The development and application of TKIs have significantly improved the prognosis of CML patients, but these drugs can only delay the progression of the disease, and cannot be used as a curative treatment (2). Due to the existence of resistance mechanisms, patients inevitably relapse (5). Therefore, it is particularly important to explore more potential therapeutic targets and markers for disease prediction and progression assessment in CML. In this study, we focused on the diagnostic markers of CML and their underlying biological mechanisms. Based on the DEGs between CML and normal samples and the CML-related genes identified by WGCNA analysis, we used LASSO regression analysis to screen out 6 hub genes (LINC01268, NME8, DMXL2, CXXC5, SCD, and FBN1).

We also focused on the co-expressed gene network identified by WGCNA analysis. The results showed that the brown module was significantly positively correlated with CML (Cor=0.39, P=7e-07). It reflects the correlation of the module as a whole with the CML phenotype. Although this correlation did not reach an exceptionally high level, a coefficient close to 0.4 suggests its reliability to some extent. Thus, it can be inferred that the brown module partially reflects gene co-expression patterns in CML transcriptome while uncovering underlying biological mechanisms. We found that the brown module genes positively correlated with CML were enriched in a variety of metabolic pathways, revealing the more active metabolic characteristics of CML cells. Several studies confirmed that targeting mitochondrial oxidative phosphorylation and glucose uptake is a potential therapeutic target for CML (20, 21). Most of the module genes negatively correlated with CML were involved in immune regulation and immune cell activation. Subsequent analysis showed that the infiltration of immune killer cells such as CD8+ T cells was significantly reduced in CML samples, confirming the immune deficiency characteristics. Cayssials et al. found that the sustained treat-free remission of CML was associated with an increased frequency of innate CD8+ T cells (22), and Harada et al. revealed that the inhibition of differentiation of dendritic cells in the hematopoietic microenvironment, as well as the up-regulation of immune checkpoint expression such as PD-L1, were responsible for the impairment of CML immune function (23). Based on this, we believe that targeted inhibition of metabolism and enhancement of immune response are important strategies for CML treatment.

It is worth noting that **Figure 1G** illustrates the association between module membership (MM) and gene significance (GS), it reflects the association of individual genes in the module with the module (x-axis, MM) and with the CML phenotype (y-axis, GS). If the correlation between MM and GS is high, the higher the correlation between the module gene and the module, the higher the correlation between the module gene and the CML phenotype, showing an overall distribution trend. We further calculated the correlation coefficient between these two types of coefficients; although Cor=0.2 with P=0.00011 indicates a weak positive relationship, it still signifies statistical significance. In this scatterplot analysis, we focused on points with strong correlations with both MM and GS. The correlation coefficients for both GS and MM of the 17 overlapping genes shared by differentially expressed genes and brown module genes were found to be greater than 0.4. Additionally, the correlation coefficients for both GS and MM of hub genes identified through LASSO regression analysis were greater than 0.5. This indicates that hub genes were significantly positively correlated with both the CML phenotype and the brown module. In this study, we utilized WGCNA analysis and LASSO regression analysis to identify hub genes of CML, and analyze their diagnostic value and potential biological functions. Therefore, WGCNA played a discriminating role to some extent. For the phenomenon that the correlation coefficients between module and phenotype and between MM and GS did not reach a high level, we believe that it may be due to the small size of CML samples included in the study. Since CML accounts for only about 15% of all leukemias, this disease is much less studied than other acute leukemias, and thus, the relevant sequencing data will be smaller. However, the two CML cohorts included in our study are currently the largest sample size cohorts with normal samples that can be found in public databases and are also representative.

The hub genes we identified are likely to be important molecules in CML metabolism and immune regulation. Stearoyl coenzyme A desaturase (SCD), a lipase that converts saturated fatty acids to monounsaturated fatty acids, is a key regulator of fatty acid metabolism pathways, its expression is also associated with poor prognosis in several cancer types (24), and elevated SCD levels also protect cancer cells from ferroptosis (25–27). Its upregulation in CML may also contribute to cancer cell growth and treatment resistance by affecting fatty acid metabolism. The high expression of LINC01268 promotes the progression of HCC by regulating MAP3K7 (28). Exosomal lncRNA LINC01268 is also a cancer-promoting factor for pancreatic cancer (29). NME/NM23 family member 8 (NME8) has been identified as a predisposition variant in breast cancer and a prognostic marker in diffuse large B-cell lymphoma (30, 31). DMXL2 has also been proposed as a potential therapeutic target for breast cancer and oral mucosal melanoma (32, 33). CXXC5 is a member of the CXXC-type zinc finger protein family. It can regulate various signal transduction processes, including TGF-β, Wnt, and ATM-p53 pathways, thereby regulating cell proliferation, differentiation, and apoptosis, and has been implicated in cancer occurrence and progression in many studies (34). Fibrillin-1 (FBN1) promotes gastric cancer progression by activating TGF-β1 and PI3K/Akt pathways, and is targeted by miR-486-5p to inhibit the growth of thyroid cancer cells (35, 36). These studies have all revealed the promoting role of hub genes in a variety of cancers, however, their relationship to CML has not been elucidated, and more in-depth mechanistic exploration is expected to reveal their role and potential value as therapeutic targets.

Moreover, we confirmed the diagnostic value of hub genes in both the analysis and validation cohorts. The risk score model constructed by LASSO regression analysis further improved the diagnostic accuracy. The discovery of these markers provides new targets for the diagnosis and treatment of CML. Finally, we identified two distinct molecular subtypes based on hub gene expression, with Cluster B having a lower risk score and infiltrating a higher proportion of CD8+ T cells and activated dendritic cells. However, the expression of immune checkpoints such as PD-L1, CTLA4, HAVCR2, and PD-1 was significantly up-regulated in Cluster B, as well as the higher TIDE score, indicating that this molecular subtype has a certain degree of immunosuppression, which inhibits the tumor-killing function of immune cells. Therefore, immunotherapy of patients in this subtype may have a higher response. In addition, drug prediction analysis showed that Cluster B was more sensitive to commonly used TKIs. The identification of molecular subtypes provides a new strategy for precise treatment of CML. Finally, we verified the expression of hub genes in larger clinical sample sizes, and confirmed that inhibition of LINC01268 expression significantly reduced CML cell viability and promoted apoptosis *in vitro*. These results reveal the oncogenic role of LINC01268 and its potential as a therapeutic target for CML. Another study showed that LINC01268, a lncRNA involved in the epigenetic regulation of AML, exerts deacetylation by directly activating HDAC2 and generating positive feedback with HDAC2. In addition, HDAC2 stimulates the transcription of LINC01268, and the expression of LINC01268 is also associated with poor prognosis and cell proliferation in AML (37). Therefore, combined with our findings, LINC01268 is most likely a malignant regulator of myeloid leukemia. However, our study also has some limitations, such as the still small size of clinical samples for the validation of diagnostic signatures and the lack of a more in-depth experimental analysis of hub genes function in CML cells. In addition, the correlation and biological mechanisms of hub genes with CML progression and drug resistance deserve further exploration, thus providing new targets for CML drug resistance treatment, which we will further refine in future studies.

## Conclusion

In summary, through WGCNA analysis and LASSO regression analysis, this study provides a better understanding of the role of biomarkers LINC01268, NME8, DMXL2, CXXC5, SCD, and FBN1, and provides a biological basis for further investigation of CML diagnosis and treatment.

## Data availability statement

The datasets presented in this study can be found in online repositories. The names of the repository/repositories and accession number(s) can be found in the article/**Supplementary Material**.

## Ethics statement

The studies involving humans were approved by ethics committee of The Second Hospital of Nanchang University. The studies were conducted in accordance with the local legislation and institutional requirements. The participants provided their written informed consent to participate in this study.

## Author contributions

FZ: Data curation, Formal analysis, Funding acquisition, Methodology, Software, Validation, Visualization, Writing – original draft. FY: Validation, Visualization, Writing – original draft. SX: Validation, Visualization, Writing – original draft. JZ: Validation, Visualization, Writing – original draft. JL: Conceptualization, Funding acquisition, Project administration, Resources, Supervision, Writing – review & editing. XW: Conceptualization, Funding acquisition, Project administration, Resources, Supervision, Writing – review & editing.
