# Supplementary material for: Identification and validation of hub genes and molecular classifications associated with chronic myeloid leukemia
Source: Front Immunol. 2024 Jan 12;14:1297886. doi: 10.3389/fimmu.2023.1297886 (PMC10811081; doi:10.3389/fimmu.2023.1297886)
Supplement: Supplementary file 1 [file Table_1.docx]

**Supplementary Table 1. Primers and siRNAs.**

| Primes and siRNAs. | |  |  | | Sequences |
| --- | --- | --- | --- | --- | --- |
| GAPDH | Forward | | | 5′-ATGGTGAAGGTCGGTGTGAA-3′ | |
|  | Reverse | | | 5′-GAGTGGAGTCATACTGGAAC-3′ | |
| LINC01268 | Forward | | | 5′-TGATCCCACTCTAGCCATCC-3′ | |
|  | Reverse | | | 5′-AAACCATCGCCAAAATCAAG-3′ | |
| NME8 | Forward | | | 5′-CAGGTGAAATGGCTCGACCT-3′ | |
|  | Reverse | | | 5′-TCGAAGTCACACTGGTCTGC-3′ | |
| DMXL2 | Forward | | | 5′-CAAGTCAGTTGTGTGGAGTGT-3′ | |
|  | Reverse | | | 5′-CCACTGGCACTTGAGTTGAC-3′ | |
| SCD | Forward | | | 5′-AAACCTGGCTTGCTGATG-3′ | |
|  | Reverse | | | 5′-GGGGGCTAATGTTCTTGTCA-3′ | |
| FBN1 | Forward | | | 5′-TTTAGCGTCCTACACGAGCC-3′ | |
|  | Reverse | | | 5′-CCATCCAGGGCAACAGTAAGC-3′ | |
| CXXC5 | Forward | | | 5′-CGGTGGACAAAAGCAACCCTAC-3′ | |
|  | Reverse | | | 5′-CGCTTCAGCATCTCTGTGGACT-3′ | |
| si-NC |  | | | ACGTGACACGTTCGGAGAA | |
| si-LINC01268 | Smart si-lencer 1 | | | GCCTATTGACCAACATGTT | |
| si-LINC01268 | Smart si-lencer 2 | | | GCAAATTCCGAAGTAGCAT | |
| si-LINC01268 | Smart si-lencer 3 | | | CCACCAGTCCCTCACATAA | |
| si-LINC01268 | Smart si-lencer 4 | | | ACATCCAATGGAGAAGAATG | |
| si-LINC01268 | Smart si-lencer 5 | | | CCAGCTATCCAAACAACTAC | |
| si-LINC01268 | Smart si-lencer 6 | | | ACCAAGTGCCAACATATACG | |

**Supplementary Table 2. Correlation of overlapping genes with phenotype and module**

| ID | moduleColor | GS.CML | p.GS.CML | MMbrown | p.MMbrown |
| --- | --- | --- | --- | --- | --- |
| LINC01268 | brown | 0.614133 | 6.41E-17 | 0.661136 | 3.34E-20 |
| NME8 | brown | 0.599483 | 5.27E-16 | 0.631405 | 4.63E-18 |
| DMXL2 | brown | 0.574064 | 1.59E-14 | 0.664353 | 1.89E-20 |
| CXXC5 | brown | -0.55167 | 2.54E-13 | -0.67036 | 6.45E-21 |
| SCD | brown | 0.549076 | 3.46E-13 | 0.628839 | 6.92E-18 |
| FBN1 | brown | 0.543144 | 6.92E-13 | 0.535954 | 1.58E-12 |
| FRMD3 | brown | 0.511423 | 2.27E-11 | 0.651438 | 1.77E-19 |
| CLC | brown | 0.505212 | 4.30E-11 | 0.73672 | 6.27E-27 |
| ADGRE3 | brown | 0.494477 | 1.27E-10 | 0.74348 | 1.21E-27 |
| PTH2R | brown | 0.475316 | 7.94E-10 | 0.544645 | 5.82E-13 |
| PROK2 | brown | 0.455252 | 4.82E-09 | 0.608541 | 1.45E-16 |
| MCTP1 | brown | 0.449921 | 7.64E-09 | 0.773715 | 3.87E-31 |
| BEX1 | brown | 0.430804 | 3.73E-08 | 0.529321 | 3.31E-12 |
| CMTM2 | brown | 0.419656 | 9.01E-08 | 0.684264 | 4.81E-22 |
| TSPAN2 | brown | 0.419378 | 9.21E-08 | 0.903709 | 2.16E-56 |
| MME | brown | 0.414475 | 1.34E-07 | 0.43288 | 3.16E-08 |
| SLC28A3 | brown | 0.40151 | 3.54E-07 | 0.648961 | 2.69E-19 |
